# Diversification in the steppe rat snake *Elaphe dione* (Pallas, 1773) coincides with the Mid-Pleistocene climatic transition of Eurasia

**DOI:** 10.7717/peerj.20351

**Published:** 2026-02-02

**Authors:** Evgeniy Simonov, Polina Chernigova, Artem Lisachov, Kazhmurat Akhmedenov, Oleg Ermakov, Anastasia Klenina, Andrey Bakiev, Roman Nazarov, Sayagul Akhmedenova, Daniel Jablonski

**Affiliations:** 1 Severtsov Institute of Ecology and Evolution, Russian Academy of Sciences, Moscow, Russia; 2 Tyumen State Medical University, Tyumen, Russia; 3 Animal Genomics and Bioresource Research Unit, Faculty of Science, Kasetsart University, Bangkok, Thailand; 4 Institute of Cytology and Genetics, Siberian Branch of the Russian Academy of Sciences, Novosibirsk, Russia; 5 Makhambet Utemisov West Kazakhstan University, Uralsk, Kazakhstan; 6 Department of Zoology and Ecology, Penza State University, Penza, Russia; 7 Samara Federal Research Scientific Center RAS, Institute of Ecology of Volga River Basin RAS, Togliatti, Russia; 8 Zoological Museum of Moscow State University, Moscow, Russia; 9 Department of Zoology, Comenius University in Bratislava, Bratislava, Slovakia

**Keywords:** Reptiles, Squamate, Colubridae, Palearctic, Biogeography, Evolution

## Abstract

**Background:**

The steppe rat snake, *Elaphe dione*, has one of the broadest terrestrial distributions among snakes. Its distribution spans from the Azov Sea and the Caucasus to the Pacific coast of Far East Asia. The steppe rat snake is one of the few reptile species with an extensive distribution in both the Western and Eastern Palearctic, making its evolutionary history of particular interest in understanding biogeographical patterns and connections between these regions. However, knowledge of its genetic variability and phylogeography remains limited. In this study, we examined the phylogeographic structure of *E. dione* to shed light on its genetic diversity and diversification history in the Western and Eastern Palearctic.

**Methods:**

We reconstructed phylogenies and analyzed the genetic structure of *E. dione* populations originating from most of its geographic range using three mitochondrial DNA gene fragments (12S rRNA, COI, ND4+tRNAs). In total, we analyzed sequences from 130 *E. dione* specimens from 100 locations. We used maximum likelihood and Bayesian inference methods to reconstruct phylogenetic trees, supplemented by an analysis of haplotype networks, molecular clocks, and a neutrality test for historical demography.

**Results:**

We identified 11 phylogeographic lineages grouped into three broader clades that diverged during the Late Miocene-Pliocene. The average uncorrected genetic distance between these 11 lineages ranged from 0.7% to 6.7% based on sequences of the COI fragment. Most of the contemporary range of *E. dione* is occupied by a single clade, with lineages distributed west and east of the Central Asian mountains. This west-east split in the clade occurred approximately 1.7 million years ago (Mya), followed by vicariant radiation in the Western and Eastern Palearctic during the Mid-Pleistocene era. Spatial patterns of mtDNA variation identified areas of post-last glacial maximum (LGM) dispersal and secondary contact zones of several lineages in the Altai and the Changbai Mountains.

**Discussion:**

Our study is the most comprehensive phylogeographic analysis of *E. dione* to date. The territory of central China most probably served as an ancestral area of this species, where *E. dione* diverged from its most recent common ancestor with *E. bimaculata* during the Late Miocene. The most active period of diversification in *E. dione* was estimated to have occurred later (∼1.3 Mya) than other widespread Palearctic species. Furthermore, this period is correlated across the species’ range and coincides with the beginning of the Mid-Pleistocene climatic transition. Climatic and environmental transitions during this period may have triggered the allopatric divergence of *E. dione* in multiple glacial refugia. Notably, diversification in the Western Palearctic resulted in a greater number of phylogeographic lineages, which could be linked to a greater number of suitable refugia. However, further evidence is needed to confirm these scenarios.

## Introduction

Cyclic climatic changes during the Pleistocene epoch exerted a strong influence on the evolutionary history of species within Eurasia (Hewitt, 1999; Stewart et al., 2010; Feliner, 2011). This influence was caused by a complex suite of environmental factors, including fluctuations in temperatures, precipitation, seasonality, and continentality (Ruddiman et al., 1989; Herbert et al., 2010; Rao et al., 2013), which changed the distributions of entire biomes (Binney et al., 2017). Traditionally, the Western Palearctic (WP) region of Eurasia, especially Europe, has been the focus of phylogeographic studies across a wide range of taxonomic groups (*e.g.*, Feliner, 2011; Speybroeck et al., 2020; Pârâu & Wink, 2021; Dapporto et al., 2024). This has led to the accumulation of phylogeographic and palaeoecological evidence suggesting that numerous faunal (and especially floral) elements may have survived glaciation periods in areas previously considered unsuitable (*e.g.*, Salvi et al., 2013; Zinenko et al., 2015; Vörös et al., 2016; Kindler, Graciá & Fritz, 2018). Similarly, these studies raise questions about how drastic the waves of biotic turnover were during the last glaciation cycles of the northern temperate regions (Schmitt & Varga, 2012; Sumegi et al., 2022; Molnár et al., 2023). However, there have been comparatively less phylogeographic studies in the Eastern Palearctic (EP) despite a recent increase in related publications (reviewed in Dufresnes & Litvinchuk, 2022; Fu & Wen, 2023). The boundary between these two regions is debatable, but here, we define the EP region as the part of Palearctic area east of the mountains of Central Asia (Hindu Kush, Pamir, Tian Shan, Dzungarian Alatau, Tarbagatai, Altai), including East and Far East Asia. The contemporary and historical climatic conditions in the WP are significantly impacted by the prevailing mid-latitude winds known as the westerlies. In contrast, the EP is influenced by monsoons, implying a distinct temperature-precipitation regime both in the past and present (Ao et al., 2021). Such conditions establish contrasting environmental backgrounds for the biogeographic histories of species inhabiting the western and eastern parts of the Palearctic. It is therefore particularly important to examine the phylogeographic patterns of species distributed across both of these regions.

The steppe rat snake *Elaphe dione* (Pallas, 1773) has a notably extensive Eurasian distribution spanning the northern coast of Azov Sea and the Donets Ridge, traversing the Caucasus and Central Asia, and extending to the Pacific coast of the Far East Asia. This species also demonstrates high ecological plasticity, allowing it to inhabit diverse habitats, including forests of various types, floodplains, meadows, steppes, the bushy slopes of gorges, salt marshes, stony and clay semi-deserts, and rocky mountain slopes. It can be also found in alpine and subalpine meadows up to 3,000–3,500 m above sea level. In addition to natural habitats, this species has also been documented in human-altered landscapes, including residential areas, gardens, and cultivated land (Bannikov et al., 1977; Kireev, 1983; Obst & Scerbak, 1993; Ananjeva et al., 1997; Bakiev et al., 2009; Kuranova et al., 2010).

Surprisingly, no comprehensive phylogeographic studies of *E. dione* have been conducted so far, and little is known about the genetic variability throughout its range. The first DNA sequence data published for this species consisted of mitochondrial DNA (mtDNA) sequences from the cytochrome *b* gene in a study by Lenk, Joger & Wink (2001), which focused on the phylogenetic relationships of all European rat snakes. In their phylogeny, the position of the *E. dione* within the genus *Elaphe* suggested this species underwent a recent expansion to Eastern Europe from the East. This study was followed by the work of Utiger et al. (2002), which focused on molecular systematics and phylogeny of *Elaphe* sensu lato. These authors sequenced several *E. dione* specimens for the cytochrome coxidase subunit I (COI) and 12S rRNA (12S) genes, and confirmed a sister relationship of *E. dione* and *E. bimaculata,* with a large genetic distance between them, validating the status of the latter species. Utiger et al. (2002) also proposed that the divergence between the two species occurred well in advance of the geographic expansion of *E. dione.* These findings were further confirmed by Burbrink & Lawson (2007) with the use of both mitochondrial and nuclear markers. Subsequent studies employing DNA barcodes in South Korea (Jeong et al., 2013) and China (Wu et al., 2023) also contributed to the understanding of local genetic variability of the species. In 2016, additional findings of *E. dione* from Western Sichuan (China) were reported (Hofmann, Fritzsche & Miehe, 2016), accompanied with sequences from several mtDNA markers that showed high intraspecific differentiation (5.1–5.8% uncorrected *p*-distances compared to other *E. dione*). It was also revealed and emphasized that some sequences previously published in GenBank (NCBI) under the name *E. bimaculata* derived from *E. dione* specimens due to species misidentification (Hofmann, Fritzsche & Miehe, 2016; Simonov et al., 2018).

Given the fact that *E. dione* has one of the broadest terrestrial distributions among snakes, we considered it essential to undertake a comprehensive phylogeographic investigation and clarify previous taxonomic confusions. Here, we aimed to provide insights into the phylogeographic structure of *E*. *dione* across its entire range using mtDNA data and to compare its genetic diversity and diversification history between the Western and Eastern parts of the Palearctic region.

## Materials & Methods

### Tissue sampling

**Figure 1:**
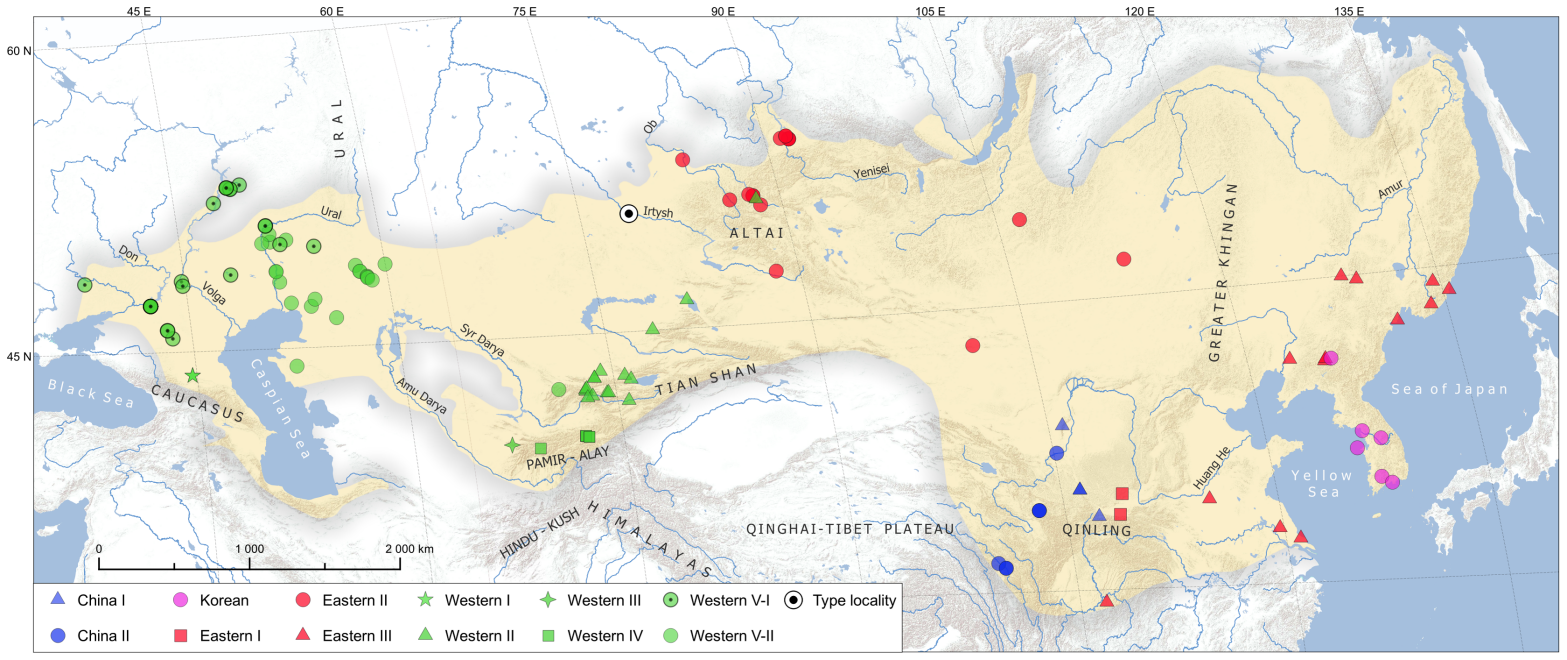
Map showing the geographic distribution of *Elaphe dione* samples included in this study overlying the yellow area, which is its approximate geographic distribution. Color and shape combinations correspond to the phylogenetic lineages recovered (see Fig. 2; locality details provided in Table S1).

Samples were obtained from museums and live collections (Zoological Museum of Moscow State University, Herpetological collection of the Institute of Ecology of the Volga River Basin of the Russian Academy of Sciences, Tula regional Exotarium) or collected in the field using minimally invasive techniques. The tissue samples consisted of buccal swabs, pieces of ventral scales, and muscles (for road-killed and museum specimens), and were preserved in 96% ethanol and stored at −25 °C. A total of 98 samples, originating from 72 locations (Fig. 1), were prepared for further sequencing and analysis. Although our samples, combined with previously published data, cover most of the species’ range, certain regions, *i.e.,* Transcaucasia, northern Iran, northern Uzbekistan, Afghanistan, and parts of central Kazakhstan, remain underrepresented due to logistical constraints.

This work primarily relied on museum and live collections. The animal study protocol and field sampling were approved by the Ethics Council of M. Utemisov West Kazakhstan University (No. 2480; date of approval 4 September 2023) for the project AP 19675960, and field permit for sampling in Uzbekistan was issued by the Ministry of Ecology, Environmental Protection and Climate Change of the Republic of Uzbekistan (No. 3310-8464-f6c5-b33e-c208-2408-8455/2023-09-05).

### DNA extraction, amplification and sequencing

DNA extraction was performed using QIAmp DNA Mini kits (QIAGEN) or by phenol-chloroform extractions (Sambrook & Russell, 2006). To complement the most widely sequenced mtDNA markers for *E. dione* in public databases, we sequenced fragments of COI (657 bp) and 12S (600 bp) genes. To amplify COI, we used the vertebrate universal primer pair: VUTF 5′-TGTAAAACGACGGCCAGTTCTCAACCAAYCAYAARGAYATYGG-3′ and VUTR 5′-CAGGAAACAGCTATGACTARACTTCTGGRTGKCCRAARAAYCA-3′ (Abramson et al., 2012). For 12S, a pair of primers originally designed for *Elaphe* were used: 12S268 5′-GTGCCAGCGACCGCGGTTACACG-3′ and 12S916 5′-GTACGCTTACCATGTTACGACTTGCCCTG-3′ (Utiger et al., 2002). In addition, some of the samples were amplified and sequenced for a fragment containing the NADH dehydrogenase four gene (ND4) plus the downstream Serine, Histidine, and Leucine tRNAs (900 bp; ND4+tRNAs), using a pair of primers ND4 5′-CACCTATGACTACCAAAAGCTCATGT AGAAGC-3′ and Leu 5′-ACCACGTTTAGGTTCATTTTCATTAC-3′ (Arevalo, Davis & Sites, 1994).

For COI, the polymerase chain reaction (PCR) protocol was a 3 min denaturation step at 95°C, followed by 35 cycles of denaturation for 30 s at 95 °C, primer annealing for 30 s at 54 °C, and extension for 1 min at 72 °C, with a final extension step at 72 °C for 5 min. For 12S rRNA, the protocol was identical, but the annealing temperature was 56 °C. To increase the productivity of the PCR for ND4, a touchdown protocol was used with the same temperatures for denaturation and elongation, but with annealing temperatures during the first seven cycles with starting at 63 °C and decreasing by 1 °C each cycle, followed by 30 cycles with annealing temperatures of 57 °C. PCR products were purified using spin columns, by extraction from polyacrylamide gel, or by using ExoSAP-IT enzymatic clean-up (USB Europe GmbH, Staufen, Germany; manufacturer’s protocol). Sequencing was performed by Macrogen Europe Inc. (Amsterdam, The Netherlands) or by the Evrogen company (Moscow, Russia) using the same set of primers as the PCRs. New sequences have been deposited in GenBank (NCBI) under accession numbers PV653725 –PV653803, PV657704 –PV657788, and PV660112 –PV660157 (Table S1).

### Phylogenetic analyses

The newly obtained sequences were manually curated using Chromas 2.6.2 (Technelysium Pty Ltd) and checked for unexpected stop codons in SeaView 4.4.2 (Gouy, Guindon & Gascuel, 2010). We combined our sequences with those available in GenBank (see Table S1) and manually aligned each marker set individually. Notably, we did not include previously published sequences of species that were incorrectly assigned as *E. bimaculata* (see Hofmann, Fritzsche & Miehe, 2016; Simonov et al., 2018, for discussion). After preliminary examination, we also excluded the sequences generated in Utiger et al. (2002) study for sample SH545 (AY122831, AY122747) due to an apparent error in the sample’s indicated origin (“Ukraine”; despite the sequences being identical to the lineage from the Russian Far East). Additionally, we excluded the data from the voucher specimen LSUMZ 45799, which originally appeared in Burbrink & Lawson (2007) with an indicated origin of “Russia”. This origin was also indicated in some subsequent publications (*e.g.*, Hofmann, Fritzsche & Miehe, 2016). However, we found that the specimen did not align with any lineages from Russia in our dataset. Subsequently, we found that the specimen is listed in the Louisiana State University Museum of Natural Science database as originating from the USSR and being “purchased from Zooherp” (DA Boyd [LSUMZ collection manager], 2025, pers. comm.).

We used the program DnaSP 6.12 (Rozas et al., 2017) to calculate the nucleotide diversity (*Pi*), number of polymorphic sites (*S*), and average number of differences (*K*) using a mode with pairwise exclusion of missing data for general characterization of DNA variation in each marker set. The number of haplotypes (*h*) was determined manually to curate the instances of shorter sequences.

For the reconstruction of the phylogenetic tree, 12S, COI and ND4+tRNAs sequences were concatenated into a supermatrix allowing for missing data. Identical individuals were detected by calculating the number of base differences per site across all sequence pairs with pairwise deletion of ambiguous/missing positions in MEGA 11 (Tamura, Stecher & Kumar, 2021) and excluded from the phylogenetic tree reconstruction. Sequences of sister species *E. bimaculata* along with *E. carinata* and *E. schrenckii* were included in phylogenetic analyses as outgroups. The best-fitting partition schemes and substitution models were selected using ModelFinder (Kalyaanamoorthy et al., 2017) based on Bayesian information criterion (BIC) score, as implemented in IQ-TREE 2.4.0 (Minh et al., 2020). For the subsequent maximum likelihood (ML) tree estimation, the full set of DNA models was considered, while for Bayesian phylogenetic inference (BI) in MrBayes v3.2.7a (Ronquist et al., 2012), the set of models was limited to those supported by the program. ML analysis was performed in IQ-TREE2 with 1,000 non-parametric bootstrap replicates under the following partitioning scheme: (1) 12S + COI codon position 1 + ND4 codon position 1 + tRNAs under the HKY+F+I model; (2) COI codon position 2 + ND4 codon position 2 under the F81+F model; (3) COI codon position 3 + ND4 codon position 3 under the TN+F model. BI in MrBayes was performed using the same partitioning scheme and models except for the HKY model for partition 3. We conducted two simultaneous runs of four Markov Chains Monte Carlo (MCMC), each run consisted of 1*10^6^ generations with a sampling frequency every 500 generations. The first 25% of generations were discarded as burn-in. The convergence of runs was assessed by examination of the average standard deviation of split frequencies and the potential scale reduction factor. Stationarity was confirmed by examining posterior probability (PP), log-likelihood, and all model parameters for effective sample sizes (ESSs > 200) and by examining trace plots of the MCMC output using the program Tracer 1.9. (Rambaut, Drummond & Suchard, 2014). Phylogenetic trees resulting from the ML and BI analyses were visualized and edited using FigTree 1.4.4. We designated phylogeographic lineages as genealogically connected organisms, that presumably maintained reticulate relationships due to reproductive connections and gene flow (*i.e.,* shallow splits), while deeper splits of ancestral lineages and all their descendants were referred to as distinct clades (Hillis, 2022; Dufresnes, Poyarkov & Jablonski, 2023).

To better resolve recent and probably non-bifurcating phylogenetic splits, we also constructed haplotype networks for samples with at least three (12S, COI, ND4+tRNAs) or two (12S, COI) sequenced markers representing two groups of phylogeographic lineages (Western and Eastern, see below) that diverged most recently and occupy most of the range of *E. dione*. To accomplish this, we generated haplotype networks using the TCS algorithm (Clement, Posada & Crandall, 2000) in PopART 1.7 software (Leigh & Bryant, 2015).

### Divergence dating

For divergence dating, we used a Bayesian approach with fossil node calibration implemented in BEAST2 v.2.7.7 (Bouckaert et al., 2019). Only the COI haplotype dataset (43 sequences, 618 bp long) was used because it was the only dataset with all identified lineages of *E. dione*. We used the bModelTest package (Bouckaert & Drummond, 2017) in BEAST2 to infer the codon partitioned nucleotide substitution models during the MCMC analysis. We adopted four ‘external’ calibration points previously established and used in divergence dating of Colubrinae (Burbrink & Lawson, 2007; Pyron & Burbrink, 2009; Chen et al., 2013; Kyriazi et al., 2013). We supplemented our sequences with available GenBank data for the taxa involved in the calibration and outgroups (see Table S1 and Fig. S1). The resulting data set consisted of 91 sequences (618 bp). Following an approach of Kuriyama et al. (2011), the prior age distributions were chosen such that the youngest age of the distribution corresponded to the youngest possible age at which this lineage existed. The following fossil calibration age constraints were set: C1 (earliest *Coluber* and *Masticophis* fossils; mean = 0.0, SD = 0.843, offset = 11.0), C2 (earliest *Lampropeltis* fossil; mean = 0.0, SD = 0.843, offset = 15.0), C3 (earliest *Pantherophis* fossil; mean = 0.0, SD = 0.843, offset = 16.0), C4 (earliest *Salvadora* fossil; mean = 0.0, SD = 0.843, offset = 20.0). See Burbrink & Lawson (2007) and Pyron & Burbrink (2009) for a detailed explanation of these calibrations. The analysis was run for 100 million generations with a sampling frequency of 10,000 generations. A relaxed uncorrelated lognormal clock model, birth–death model of speciation, and random starting tree were applied. The analysis was repeated four times and parameter log files, and the phylogenetic trees were combined using LogCombiner 2.7.7 with a burn-in of 10% for each replicate. To assess the convergence, stationarity, and effective sample sizes (for ESSs >200) for all parameters, we used Tracer 1.7.1 (Rambaut et al., 2018). The maximum clade credibility tree with mean node heights was calculated in TreeAnnotator 2.7.7. The tree was visualized using FigTree 1.4.4.

### Diversity and historical demography

For each phylogenetic unit of *E. dione* with sufficient sample size, we tested for deviations from neutrality under a constant population size model. We performed three neutrality tests in DnaSP: Fu’s *Fs* (Fu, 1997), R_2_ (Ramos-Onsins & Rozas, 2002), and Tajima’s *D* (Tajima, 1989). We obtained the significance of the tests by comparing them to a null distribution of 10,000 coalescent simulations under a constant population size model. Large negative *Fs* values, small positive R_2_values, and negative *D* values can be interpreted as signatures of population expansion. We also calculated a set of genetic polymorphism statistics, including *Pi, h*, *K* haplotype diversity (*Hd*). We used the COI dataset for the calculations due to its completeness across phylogenetic groups and its high level of variation compared to 12S. Additionally, we excluded sequences with more than 10% of missing sites. However, the inclusion of other sequences with minor amounts of missing data still resulted in reduced estimations of *h* and *Hd* for some groups.

Mean uncorrected pairwise distances (*p*-distances) between revealed phylogeographic lineages and maximum distances within lineages with pairwise deletion of ambiguous/missing positions were calculated in MEGA 11 using the same dataset employed for phylogenetic tree estimations. Since COI is widely used as a DNA barcode marker for reptiles, we also calculated the average distances between the lineages for the COI data set only.

## Results

### Overall genetic variation

A total of 210 sequences were newly obtained for 98 individuals of steppe rat snake from 72 locations (Table S1). The final concatenated dataset, which incorporated both new sequences and previously published GenBank sequences, encompassed a total of 130 individuals from 100 locations. Among these, COI barcode sequences were available for 110 individuals, 12S for 103 individuals, and ND4+tRNA for 47 individuals. Summary statistics on variation of each marker are given in Table 1. The pairwise uncorrected distances for COI ranged from 0% to 7.3%, with a mean distance (±standard error (SE)) of 2.3 ± 0.3%. Pairwise distances for ND4+tRNAs ranged from 0% to 4.4% with a mean distance of 0.9 ± 0.2% and for 12S they ranged from 0% to 2.9% with a mean distance of 0.9 ± 0.2%. These estimates should be interpreted with caution because of unequal sample sizes and missing data.

### Phylogeny and phylogeography

**Figure 2:**
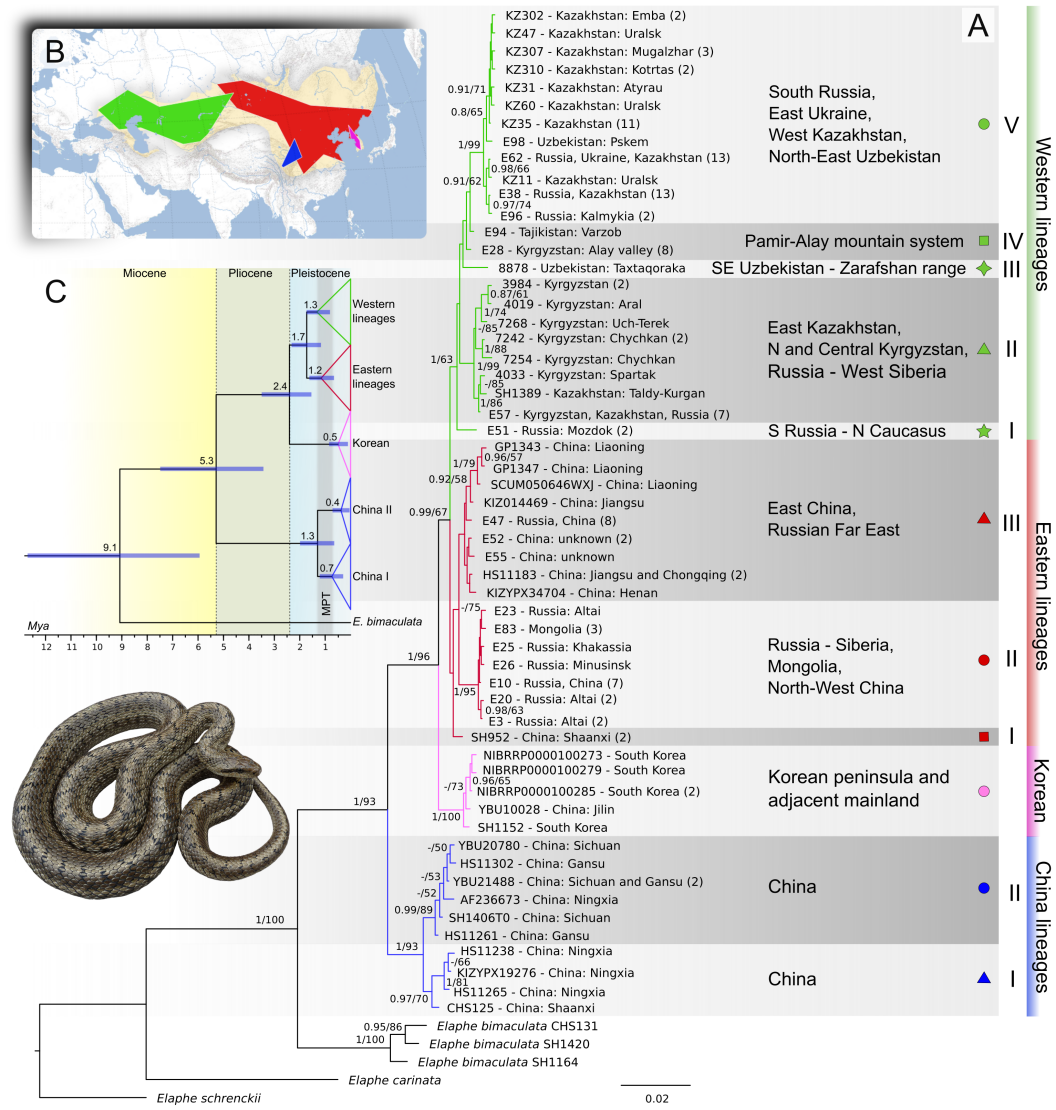
Phylogenetic relationships of *Elaphe dione*. (A) Bayesian tree reconstructed from the concatenated mtDNA dataset of unique haplotypes only (12S+COI+ND4, 2,000 bp). The major geographic groups of phylogenetic lineages are highlighted in different colors, while the lineages themselves are identified by color/shape combinations. Numbers near the branches indicate posterior probabilities greater than 0.8 and nonparametric bootstrap support values greater than 50%. Each terminal branch represents a unique haplotype with the reference sample ID, the name of the country or region where the haplotype was found, and if the haplotype was observed in more than one sample, the corresponding number of samples is given in parentheses (see Table S1 for details). Scale bar and branch lengths are given in expected substitutions per site. (B) Geographic distribution of major clades showed as polygons connected sampling localities (also see Fig. 1). (C) Time divergences based on the molecular clock analysis of COI. Numbers at nodes represent the expected time of the divergence in million years ago (Mya). The extent of the Mid-Pleistocene climatic transition (MPT) is highlighted. The pictured specimen originates from the Samara Region, Russia (Photo by A. Klenina).

The concatenated alignment used to reconstruct phylogenetic trees was 2,000 bp long containing 56 unique haplotypes of *E. dione* (Table S1). This number of haplotypes might be an underestimation due to missing markers/sites in parts of samples. Phylogenetic analysis using ML and BI estimations revealed mainly concordant trees with three major, well-supported clades (Fig. 2). The first major clade was recovered sister to all other samples of *E. dione* and contained of two lineages named China I and China II, which consists of samples exclusively from China. The China I lineage with four haplotypes had moderate support (BI posterior probability/non-parametric bootstrap: 0.97/70%) and is found in Ningxia and Shaanxi Provinces, while the China II lineage (six haplotypes, 0.99/89) is distributed in the mountains of Sichuan, south of Gansu and Ningxia Provinces (Fig. 1). The second major clade consists of a single lineage with five haplotypes, herein named Korean as its geographic distribution is limited to the Korean Peninsula and adjacent mainland China. The third major clade encompasses most of the range of *E. dione*, comprising two geographical groups of lineages: Western and Eastern. The former group, consisting of five lineages, is found west of the mountains of Central Asia, while the latter, consisting of three lineages, is mainly found east of there. The designation of these geographic groups as distinct clades is precluded by low or no support for the Eastern group of lineages in both analyses, while the clade of both groups has high PP support (0.99) and low bootstrap support (67).

**Table 1:** Genetic diversity estimates for each mtDNA marker.

**Marker**	***N*** ** (** ***n*** ** sites)**	***h***	***Pi***	***S***	***K***
*12S*	103 (552.1)	21	0.0092	34	5.0
*COI*	110 (572.8)	45	0.0230	88	12.8
ND4+tRNAs	47 (812.5)	13	0.0092	49	7.3

**Notes.**

N sample size (n sites - average number of sites analyzed) h number of haplotypes Pi nucleotide diversity (per site) S number of polymorphic sites K average number of differences

**Figure 3:**
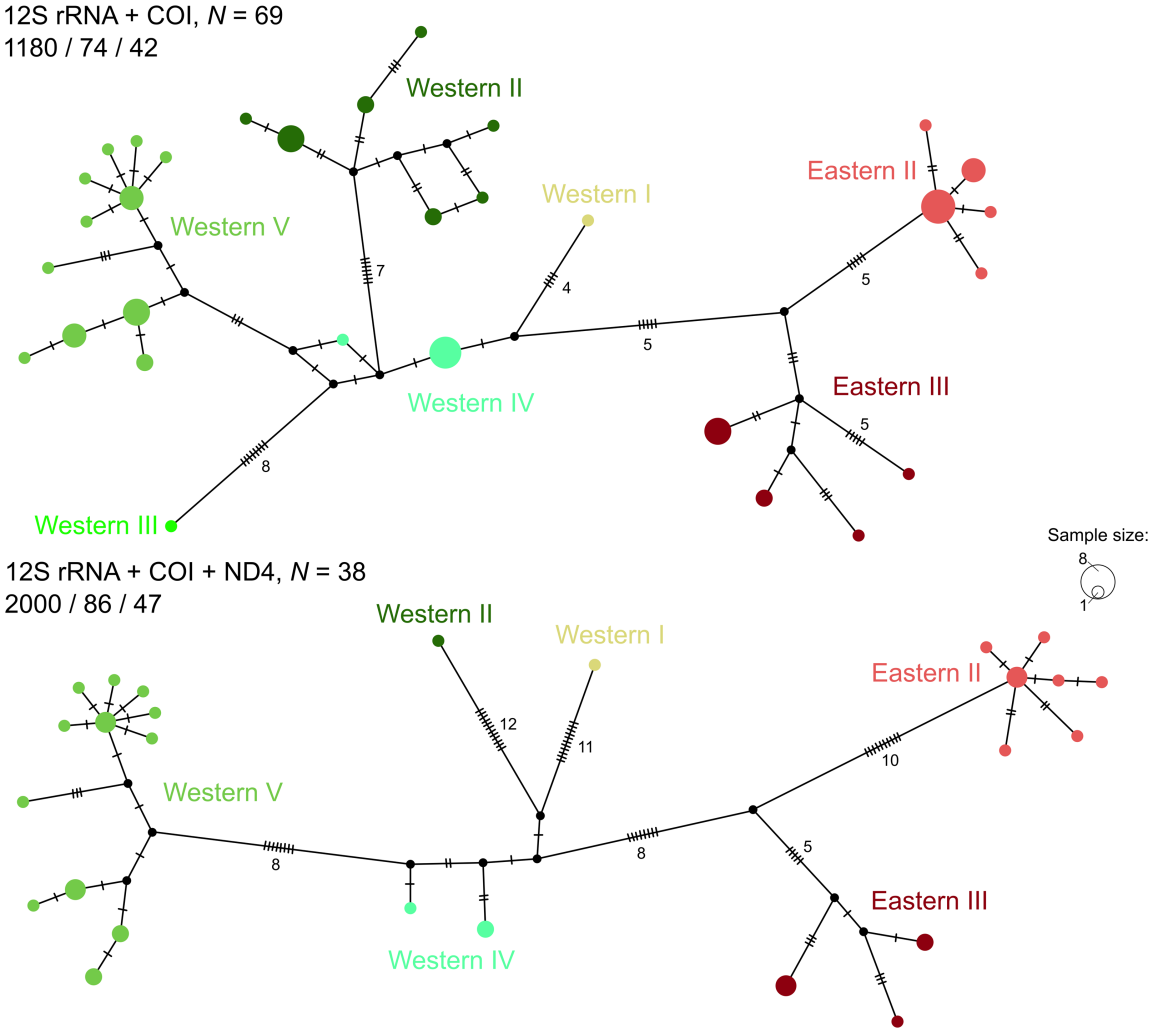
TCS haplotype networks based on the 12S+COI dataset (top) and the 12S+COI+ND4 dataset (bottom) for the lineages comprising the widespread clade of *Elaphe dione.* For each network, the sample size (N), total number of sites, number of segregating sites, and number of parsimony-informative sites are indicated. Circle sizes are proportional to haplotype frequencies. Hatch marks on connecting lines represent the number of mutational steps between haplotypes, while black circles denote inferred but unsampled intermediate haplotypes. The colors of the haplotypes correspond to defined lineages (shades of green for Western lineages and shades of red for Eastern lineages), and the lineage names match those in Figs. 1 and 2.

The Eastern I lineage is represented by two specimens of identical haplotype from the Wei River valley (central Shaanxi Province, China), which were recovered as sister to both Eastern II and Eastern III lineages with poor support. The Eastern II lineage with seven haplotypes (17 samples) had strong support (1.0/95), and its geographic distribution extends from western Mongolia to the southeast portion of the West Siberian Plain in Russia, covering the Sayan and Altai Mountains (including Xinjiang, China). This lineage formed a star-like pattern in both haplotype networks, implying fast range/demographic expansion. The Greater Khingan mountains in northeastern China possibly act as a geographical boundary between this lineage and its presumably sister lineage (moderate support 75 in ML), Eastern III. The Eastern III consisted of nine haplotypes (18 samples) whose monophyly was not supported in ML and BI analyses. Nevertheless, the members of the Eastern III group were found to form a group of a common ancestry when analyzed using haplotype networks (Fig. 3). This lineage is the most geographically widespread of all *E. dione* lineages and is found from the upper reaches of the Yangtze River (Chongqing municipality) to eastern and northeastern China regions, as well as the entire Pacific coast of the Russian Far East. In Liaoning Province, China, this lineage was found to meet the Korean lineage (Fig. 1). Two haplotypes from three snakes of Chinese origin sampled in Tula Exotarium turned out to be the members of the Eastern III lineage.

The interrelationships of the Western lineages remained unresolved in our analyses, with a strong support for their monophyly in BI and only weak support in ML (1.0/63). The Western I lineage was represented by the only sample from the Caucasian region in our dataset (North Caucasus, Russia), which was recovered sister relative to all other western lineages in the BI tree, but swapped positions with the Western III lineage (represented by a single sample from the Zarafshan Range in southeastern Uzbekistan) in the ML tree. The Western II lineage, represented by eight haplotypes (16 samples), was strongly supported (1.0/99) and distributed in northern and central Kyrgyzstan, eastern Kazakhstan and penetrating to Altai Mountains in southern Siberia (Russia) where it meets the Eastern II lineage (see Fig. 1). Under the name Western IV, we defined a group of two haplotypes (nine samples) from the Pamir-Alay Mountain system of south Kyrgyzstan (Alay valley) and Tajikistan (Varzob valley). These haplotypes were found to be paraphyletic in the trees (with no support). However, in both haplotype networks, they were placed in a “core” position relative to all other lineages of the western clade (Fig. 3) and had the smallest pairwise genetic distances relative to all other western lineages (Table 2). Thus, the haplotypes of these lineages might be ancestral to the remaining western clade. The Western V lineage is most geographically widespread in the western part of the species range, inhabiting south-east of the East European Plain, southern Urals, western regions of Kazakhstan and parts of Uzbekistan (found in Pskem river valley in west Tien Shan mountains). This lineage had strong support (1.0/99%), with 12 haplotypes identified in 51 individuals. Within this lineage, two subclades with weak to moderate support were reconstructed in both phylogenetic trees and haplotype networks (Figs. 2–3). One of them occupies the area northwest of the Caspian Sea (Western V-I), while the other occupies the area northeast of the Caspian Sea (Western V-II), with a contact in an area along the Ural River.

**Table 2:** Average uncorrected genetic distances (*p*-distance, %) between phylogeographic lineages of *Elaphe dione*. The distances based on the concatenated mtDNA dataset are given below the diagonal, while the *COI*-based distances are given above the diagonal. In diagonal (bold) are the maximum/average intraclade *p*-distances based on *COI*. All ambiguous positions were removed for each sequence pair (pairwise deletion option).

**Lineage**	**China I**	**China II**	**Korean**	**Eastern I**	**Eastern II**	**Eastern III**	**Western I**	**Western II**	**Western III**	**Western IV**	**Western V**
China I	**1.1/0.6**	1.5	6	5.6	6	5.7	6.1	6.7	5.9	5.8	6.1
China II	1.5	**0.4/0.1**	6	5.8	5.8	5.5	5.8	6.6	6.1	5.6	6
Korean	5.6	4.7	**0.6/0.3**	2.4	2.7	2	2.9	2.7	2.7	2.3	3.2
Eastern I	5.3	5	1.8	**0.0/0.0**	1.3	1	1.6	2	1.6	1.4	2
Eastern II	5.5	4.5	1.9	1	**0.6/0.1**	0.9	1.7	2	2.2	1.4	2.2
Eastern III	5.4	4.7	1.7	1	0.9	**1.5/0.6**	1.5	1.8	1.9	1.2	2
Western I	5	3.9	1.2	0.9	1.3	1.2	**NA**	1.7	1.8	0.7	1.5
Western II	6.1	4.9	2.1	1.7	1.6	1.7	1.1	**1.2/0.6**	1.8	1.1	2
Western III	5.5	4.9	1.8	1.4	1.6	1.7	1	1.5	**NA**	1.1	1.5
Western IV	5.4	4.4	1.5	1	1	1	0.5	0.9	0.8	**0.3/0.1**	0.8
Western V	5.4	4.6	2.2	1.5	1.6	1.6	1.1	1.4	1.2	0.6	**1.0/0.3**

In total, we identified 11 phylogeographic lineages. The correspondence of lineages to geographic populations are presented on Fig. 1. The average uncorrected genetic distances (*p*-distance) between lineages ranged from 0.5 (Western I *vs.* Western IV) to 6.1% (China I *vs.* Western II) in the concatenated mtDNA dataset, and from 0.7 to 6.7% in the COI (DNA barcode marker) dataset (Table 2). The highest intralineage distance of 1.5% was observed in the Eastern III lineage.

### Divergence dating and neutrality tests

Molecular dating estimates placed the divergence between *E*. *dione* and *E*. *bimaculata* stem branches in the late Miocene at 9.1 Mya (12.7–5.9 Mya of 95% highest posterior density - HPD). The first split of the ancestral lineage of China clade from the most recent common ancestor (MRCA) of *E. dione* occurred at 5.3 Mya (7.5–3.4), followed by separation of common ancestor of the Korean clade at 2.4 Mya (3.5–1.5), and the remaining split of Eastern and Western lineages within widespread clade at 1.7 Mya (2.4–1.1). Further diversification within the major clades occurred simultaneously in the Mid-Pleistocene (Fig. 2C, Table 3). Notably, the separation of the two Western V haplogroups predated the last Glacial Maximum (LGM) by 0.47 (0.73–0.24).

**Table 3:** Summary diversity statistics, neutrality tests and tMRCA for different subsets of *Elaphe dione* (with sample size >3) using COI dataset. Statistically significant values (*p* < 0.05) of neutrality tests are in bold.

**Group**	***N***	***h***	***Hd***	***Pi***	***K***	**Fu’s** ***F*** **s**	***R***_**2**_	**Tajima’s** ***D***	**tMRCA**
Overall	104	43	0.96	0.0217	11.8	**−8.57**	0.07	−0.80	5.3 (7.5–3.4)
*China clade*	9	7	0.92	0.0107	5.9	−0.85	0.20	1.10	1.3 (2.1–0.6)
China I	4	4	1.00	0.0058	3.2	−1.16	0.28	−0.31	0.7 (1.3–0.3)
China II	5	3	0.70	0.0015	0.8	−0.83	0.25	−0.97	0.4 (0.8–0.1)
*Korean clade*	5	4	0.90	0.0026	1.6	**−1.41**	** 0.19**	−1.09	0.5 (1.0–0.1)
*Widespread clade*	90	32	0.94	0.0133	7.3	**−7.66**	0.07	−0.89	1.7 (2.4–1.1)
*Eastern lineages*	32	14	0.83	0.0074	4.1	−3.26	0.09	−0.90	1.2 (1.8–0.7)
Eastern II	15	4	0.37	0.0011	0.67	−1.22	0.14	**−1.91**	0.5 (0.8–0.2)
Eastern III	16	9	0.86	0.0064	3.5	−1.98	0.13	−0.11	0.8 (1.3–0.5)
*Western lineages*	58	19	0.91	0.0101	6.1	−2.09	0.08	−0.63	1.3 (1.9–0.9)
Western II	12	5	0.76	0.0058	3.5	1.41	0.16	0.39	0.6 (1.0–0.3)
Western IV	8	2	0.25	0.0008	0.5	0.76	0.33	−1.31	NA
Western V	35	10	0.83	0.0034	2.1	−2.51	0.09	−0.87	0.5 (0.7–0.2)
Haplogroup V-I	15	4	0.70	0.0015	0.9	−0.53	0.15	−0.03	0.3 (0.5–0.1)
Haplogroup V-II	20	6	0.64	0.0017	1.1	**−2.06**	0.12	**−1.77**	0.3 (0.5–0.1)

**Notes.**

N sample size h number of haplotypes Hd haplotype diversity Pi nucleotide diversity per site *R*_2_ Ramos-Onsins & Rozas’s *R*_2_

In most cases, the null hypothesis of a constant population size within the examined subsets of the species was not rejected (Table 3). Fs was significantly negative for the entire species, the Korean lineage, the clade of Eastern+Western lineages, and the Western lineage haplogroups V-II and II. The Korean lineage also had a significant, small and positive value in the R2 test. Tajima’s D was significantly negative for the Eastern II lineage and the Western lineage of haplogroup V-II.

## Discussion

### Species origin and phylogeography

Using a molecular clock approach, we dated the split between *E. dione* and *E. bimaculata* to the late Miocene, within a wide confidence interval of 12.7–5.9 Mya (mean of 9.1 Mya). This estimate is like those in other colubrid studies that included both *E. dione* and *E. bimaculata* (Kuriyama et al., 2011; Jablonski et al., 2023), but younger than the estimate of 16.3 Mya in Burbrink & Lawson (2007). In that study, divergences between other *Elaphe* species were also older. This discrepancy may be due to the selection of calibration points and prior age distributions for the analysis (see Kuriyama et al., 2011 for discussion). Based on the current distribution of both species and the spatial patterns of genetic variation in *E. dione*, we hypothesize that their divergence might be linked to the formation of ridge-basin systems in the western Qinling Mountains by 10.5 Mya, accompanied by overall deformation of Tibetan Plateau in the mid-to-late Miocene (Ge et al., 2012), although this connection remains hypothetical and requires testing through spatial-genetic models. The Qinling Mountains, an east–west oriented range in central China, extend for over 1,500 km with an average elevation of 2,000–3,000 m. They form a major climatic boundary between northern and southern China: the south is characterized by a warm, humid subtropical climate, while the north experiences a cooler, drier temperate climate (Rost, 1994). It corresponds with the distribution of these snakes, where the present range of *E. bimaculata* lies mainly south of the Qinling, whereas *E. dione* is found primarily to the north. The range was shown to serve as a strong isolating barrier for other ectothermic vertebrates, such as fish (Shao et al., 2019; Chen, Jiao & Ni, 2022), salamanders (Huang et al., 2017) and frogs (Hu & Jiang, 2018; Othman et al., 2022).

Notably, the earliest known fossils related to *E. dione*, identified as *E.* aff. *dione*, date to the late middle Miocene (13–12 Mya) in the upper reaches of the Irtysh River in eastern Kazakhstan (Ivanov et al., 2019). This fossil may represent a species in the lineage leading to *E. dione* or the common ancestor of *E. dione* and *E. bimaculata*, taking into account our estimate of the split of these species (12.7–5.9 Mya). The occurrence of this *E.* aff. *dione* in western Central Asia is consistent with the pattern observed in other ectothermic taxa from that period (*e.g.*, indeterminate Crotalinae in the early late Miocene of Ukraine, 11.1 Mya; Ivanov, 1999). However, this was followed by a decrease in herpetofaunal diversity towards the end of the Neogene and Quaternary, which was possibly linked to progressive global cooling in the Northern Hemisphere (Vasilyan, Zazhigin & Böhme, 2017).

**Figure 4:**
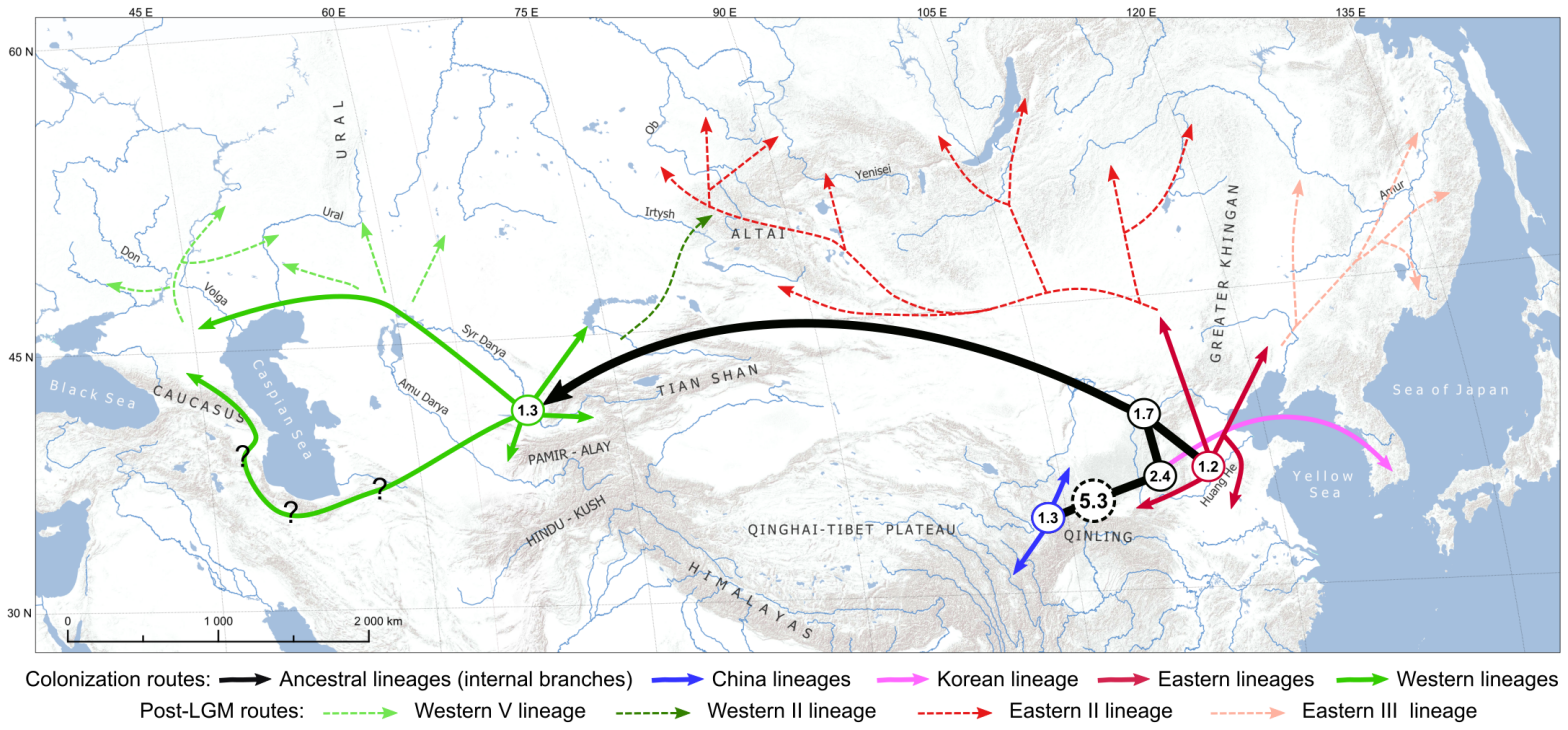
Schematic illustration of the geographical and temporal scope of the primary cladogenesis events of *Elaphe dione*. All locations are approximate. The numbers within the circles represent the time (Mya) of the most recent common ancestor. The colors correspond to those depicted in Figs. 1–3. Solid arrows indicate hypothetical directions of phylogenetic lineage colonization routes. Dashed arrows represent hypothetical post-Last Glacial Maximum (LGM) northward dispersal.

Within the species, we delineated three major clades with 11 lineages coinciding with known geographical distribution (Figs. 1 and 2). The region corresponding to present-day central China most probably served as an ancestral area of the species (Fig. 4). According to our hypothesis, the initial split around 5.3 Mya resulted in the separation of the ancestral lineage of China clade and coincided with complex climatic and environmental changes during the Miocene-Pleistocene transition (Herbert et al., 2016; Holbourn et al., 2018). The split of the ancestral lineage resulted in Korean clade dates back to the Plio-Pleistocene boundary, about 2.4 Mya, which was marked by the onset of Northern Hemisphere glaciations and a global shift to cooler climatic conditions (De Schepper et al., 2013). The Korean Peninsula is widely recognized as the most prominent and significant glacial refugium in northern East Asia (Fu & Wen, 2023). Of particular interest are the cases of similar divergence times of Korean mtDNA lineages in the Japanese tree frog, *Dryophytes japonicus* (∼2.0 Mya; Dufresnes et al., 2016) and *Pelophylax nigromaculata* sensu lato (∼2.4 Mya in Komaki et al. 2015, but ∼4.0 in Dufresnes et al. 2024), whose isolation may be linked to the same past environmental processes. The present-day representatives of the Korean clade of *E. dione* are “locked” in the peninsula, though land connections existed during the glaciation cycles, as supported by sea-level reconstructions. Notwithstanding the presumably long isolation history, the tMRCA of the modern haplotypes of the Korean clade date back to 0.5 Mya (1.0–0.1), and the significant values of Fu’s Fs and R_2_ tests (Table 3), suggest recent population expansion. This may reflect a founder effect following a population bottleneck, although demographic modelling would be needed to confirm this. The descendants of the ancestral lineage that is sister to the Korean underwent further allopatric subdivision and occupy most of the modern species’ range.

### Mid-Pleistocene diversification

The estimated west-east split of the widespread Eastern+Western clade of the steppe rat snake occurred approximately 1.7 million years ago, followed by a vicariant radiation in the WP and EP during the Mid-Pleistocene (Fig. 4). It was not possible to ascertain the interrelationships between lineages in both western and eastern groups. However, short internal branches in the ultra-metric tree might reflect a near-simultaneous divergence from a common ancestor in multiple small refugia, but this hypothesis remains to be formally tested. The mean time of the MRCA for the Western lineages is estimated at 1.3 Mya, and 1.2 Mya for the Eastern group, although monophyly of the latter was not supported. Interestingly, the split between the China I and China II lineages within the China clade also dates to the mean time of 1.3 Mya (Fig. 2C, Table 3). Thus, the time of most active cladogenesis is correlated across the species range and coincides with the beginning of the Mid-Pleistocene climatic transition (MPT; 1.25–0.7 Mya) (Fig. 2C). The MPT marks the change in the dominant periodicity of glacial–interglacial cycles, which lengthened from around 40 Kyr to around 100 Kyr with a larger amplitude (Pisias & Moore, 1981). It has been proposed that the Earth underwent a transition between “mild glacial” and “interglacial” states prior to the MPT and subsequently entered a phase of oscillation between “full glacial” and “interglacial” states (Ashwin & Ditlevsen, 2015). The reasons for this change remain debatable, and various mechanisms have been suggested (Berends et al., 2021; Shackleton et al., 2023). In a recent study, Zan et al. (2024) demonstrated pronounced landscape transformation by climatic factors around the MPT in Central Asia and northwest China using detailed paleoclimate records. The authors linked the dispersal of hominins in the Palearctic region to the widespread formation of open habitats, river terraces, and desert-loess landscapes, which occurred due to increased climate amplitude and aridity fluctuations over the MPT.

Climatic and environmental transitions during the MPT may have triggered allopatric divergence of *E*. *dione* in multiple glacial refugia, although further evidence is needed to confirm this scenario. A similar process has been suggested for many other reptiles (*e.g.*, Zinenko et al., 2015; Stümpel et al., 2016; Kindler, Graciá & Fritz, 2018; Jablonski et al., 2024); however, the timing of this subdivision within *E. dione* does not fully align with diversification observed in the few other snake species with a broad distribution in the Palearctic. Interestingly, the geographic distribution and overall pattern of *E. dione* phylogeographic subdivision resemble pit-vipers of the *Gloydius halys* species group. The steppe rat snake is often not only sympatric, but syntopic with pit-vipers, demonstrating color and behavioral mimicry of them (Obst & Scerbak, 1993). The ancestral area of these pit-vipers was also inferred to be central China (Asadi et al., 2019). Yet, the most active geographic diversification in this group is estimated to have occurred around the Plio–Pleistocene boundary and led to the phylogenetic lineages currently recognized as separate species. Further examples include the dice snake, *Natrix tessellata*, and the green toads of the *Bufotes viridis* complex, both widespread throughout the WP. In areas where their ranges overlap with that of *E. dione*, clade diversification coincides primarily with the Plio-Pleistocene boundary (2.5–1.9 Mya) as well (Dufresnes et al., 2019; Jablonski et al., 2024) suggesting similar phylogeographic scenarios due to same environmental conditions. In the EP, allopatric subdivisions in tree frogs of the *Hyla japonica* group mainly dated to the same interval (Dufresnes et al., 2016).

It remains unclear why clade diversification in *E*. *dione* occurred later than in other widespread Palearctic species. The rates of molecular clocks for COI and the divergence of the other species included in our analysis (see Fig. S1) are consistent with those published in recent studies (Kuriyama et al., 2011; Kyriazi et al., 2013; Jablonski et al., 2023). Furthermore, the raw genetic distances between lineages are shallow (see Table 2), which is reflected in the time-tree divergences. Due to the identical timing of this process throughout the almost whole species’ range, it is also implausible to assume that certain regions (*e.g.*, Central Asia) were not colonized earlier. Therefore, one possibility is that their ecological plasticity and cold tolerance delayed population isolation until more severe Mid-Pleistocene glaciations. For example, active phylogeographic subdivision in other cold-tolerant, wide-ranging Palearctic snake species in the *Vipera ursinii*/*V. renardi* complex also began with and continued after the MPT (Zinenko et al., 2015). Alternatively, the species’ distribution prior to the MPT was exceedingly limited, and the Mid-Pleistocene environmental transformations previously discussed had prompted the species’ dispersal.

### Dispersal to Eastern Europe

A particularly intriguing question in the phylogeography of *E. dione* pertains to the route by which this Asiatic species reached Europe. Based on the present distribution of the species, two hypotheses can be formulated: (i) a northern route across the north Central Asiatic plains through the territory north of the Caspian Sea followed by southward dispersal, and (ii) a southern route from Central Asia to the Alborz Mountains and Transcaucasia with subsequent northward dispersal. The position of all samples examined from the European part of the range within a group of western lineages is unambiguous. Our research has revealed that most of the samples belong to the Western V lineage, which is distributed across vast regions surrounding the northern Caspian Sea, in the southern Ural Mountains, and the Mugodzhar range. The easternmost location of a distinct haplotype of this lineage has been found in the Pskem Mountains of Uzbekistan. This finding suggests a possibly continuous distribution of the Western V lineage along the Syr Darya river valley and a Central Asian origin of this lineage. This phylogeographic pattern is consistent with a northern colonization route, although further sampling in intermediate areas is required to confirm this. Furthermore, the Western V lineage is subdivided into two haplogroups (Figs. 2 and 3), which demonstrate a clearly readable geographic pattern of subdivision along the Caspian lowland. Haplogroup Western V-I is distributed almost exclusively west of the Ural River and occupies most of the European part of the modern species’ range. The split of these haplogroups dated back to 0.5 Mya (0.7–0.2), thus significantly predating the LGM (0.026–0.019 Mya, Clark et al., 2009). The haplogroups also exhibited a distinct demographic history. Haplogroup Western V-II has signatures of population expansion/growth in the neutrality test (Table 3) and a star-like pattern of haplotypes in the network (Fig. 3). Therefore, we argue that the range expansion of the Western V-I haplogroup south of the East European plain predated the LGM, and that this population did not experience a recent expansion/growth. *Elaphe dione* may have survived in a glacial refugium or series of micro-refugia in the northern Ponto-Caspian region, as has been suggested for *Z. vivipara*, *V. berus* (Guillon et al., 2024), *V. renardi* (Zinenko et al., 2015), *N. tessellata* (Jablonski et al., 2024) and *N. natrix* (Simonov et al., 2024).

Given the phylogeographic patterns described above, it was unexpected to find that two samples from the North Caucasus region (Russia) exhibited a distinct lineage, designated as Western I (see Figs. 1–3). Considering the dense sampling in the areas north of it, we hypothesize that this lineage originated in the Caucasian/Transcaucasian region, assuming that northern Iran, Transcaucasia, and Caucasus were colonized by *E. dione via* the southern route including the area of northern Afghanistan (Fig. 4). This hypothesis warrants further examination through sampling in additional regions with a particular emphasis on Transcaucasia and the Alborz Mountains. A similar dispersal corridor between the Caucasus and Central Asia has been previously established for other species, including snakes (*Gloydius caucasicus*, Asadi et al., 2019; *N. tessellata*, Jablonski et al., 2024) and lizards (*Pseudopus apodus*, Jandzik et al., 2018). Early zoogeographical studies suggest that *E. dione* colonized Transcaucasia from the North, moving along the Caspian Sea coast (Sobolevsky, 1929a). Recently, it was acknowledged that the Turanian region served as the possible entry for Asian genera, such as *Elaphe*, into northeastern Iran; however, no specific colonization hypothesis was formulated (Moradi et al., 2024).

### Post-LGM expansion and secondary contacts

The “expansion–contraction” model of Pleistocene biogeography (Provan & Bennett, 2008) posits northward post-glacial colonization by *E. dione.* We found that only four out of 11 lineages advanced toward northern areas and occupied most of the current species’ range (Figs. 1 and 4). Accordingly, we observed low haplotype diversity in presumably recolonized areas for lineages Eastern II, Eastern III, Western II, and Western V, though the hypothesis of constant population size was not rejected for all of them in the neutrality tests (Table 3). Available fossil data indicate a wider distribution of the steppe rat snake during the early and middle Holocene in the Urals and Siberia (Ratnikov, 2009; Danukalova et al., 2014; Yakovleva & Yakovlev, 2017). Consequently, the species range underwent a contraction relative to the most optimal period after the LGM. Fossils of *E. dione* and *E.* cf. *dione* located well outside of the contemporary distribution are also known from the interglacial periods of the Lower Neo-Pleistocene (0.7–0.35 Mya) in the East European Plain (Ratnikov, 2009).

According to our data, the postglacial expansion of *E. dione* resulted in secondary contact zones between lineages, namely between the Western II and Eastern II lineages in the Altai Mountains of southern Siberia and between the Korean and Eastern III lineages in the Changbai Mountains of northeastern China. Admixture between previously isolated lineages of this species likely occurs in these areas, as is commonly observed in other species (*e.g.*, Zhang et al., 2008; Ding et al., 2011; Dufresnes et al., 2024), and this should be considered in future research. It was suggested by Fu & Wen (2023) that survival in multiple refugia followed by expansion and secondary contact is likely to be common in central and eastern China. It was also suggested that this may serve as an important mechanism for maintaining genetic diversity and a stable effective population size. We predict the existence of other contact zones between lineages of the steppe rat snake, especially in the Central Asian region, which could be elucidated through denser sampling.

#### Taxonomic implications

The only subspecies of *E. dione* considered valid by some authors (Ananjeva et al., 2004; Tuniyev et al., 2009; Vedmederya, Zinenko & Barabanov, 2009) is *E. d. czerskii* (Nikolsky, 1914). In that year, A. M. Nikolsky described *Coluber czerskii* from the Tumen River, which borders modern-day North Korea and Russia (Khasansky District of Primorsky Krai). This form was distinguished by its head shape and tail length. Later studies (*e.g.*, Obst & Scerbak, 1993) mentioned that this morphotype is common in the Russian Far East and is characterized by a reduced number of ventral scales, and is best represented as distinct subspecies.

Our results indicate that individuals from this region belong to the Eastern III lineage, which shows shallow genetic divergence (0.9−1.0% in *COI*) from its presumed sister lineages. Recognizing this lineage as a valid subspecies would require a reevaluation of all other lineages within the same taxonomic rank. We therefore propose preliminary reserving the name *E. d. czerskii* for the eastern group of lineages, contingent on future confirmation of their monophyly relative to other clades. However, the issue is further complicated by the geographical proximity of both western and eastern lineages to the species’ type locality (“Gratscheffskoi outpost, near Semijarsk, upper Irtysh area, Semipalatinsk district”) which was restricted to Grachi village in the Beskaragay district of East Kazakhstan (Mertens & Müller, 1928; see Fig. 1). Thus, it remains unclear whether the Western II or Eastern II lineage occupies this region.

Several other names of the subspecific rank have been proposed within *E. dione*. Obst & Scerbak (1993) listed *E. d. niger* Golubeva, 1923, and *E. d. tenebrosa* Sobolevsky, 1929, names that were subsequently repeated in the literature and databases (*e.g.*, Ananjeva et al., 2004; Tuniyev et al., 2009; Krymov, 2016; Simonov et al., 2018). In our view, assigning subspecific status to these forms is taxonomically unjustified for reasons explained below.

Golubeva (1923) p.4 described a “special black race [особая  черная раса] *niger*, nom. nov.” of *Coluber dione* from the vicinity of the villages of Vylkovo and Kluchevoe (modern-day Altai Krai, southwest of West Siberia, Russia), based on dark or black coloration. For unclear reasons, she used the designation “nomen novum”, which implies the replacement name rather than a new description. Obst & Scerbak (1993) added further inaccuracies by omitting parentheses around the author’s name and vaguely citing the locality as “Tomsk Region (“Gebiet von Tomsk, West-Siberien)”. Sobolevsky (1929b) described a similar unpatterned brown form from Sennoe village in the Bukhtarma River valley of the Altai Mountains (now East Kazakhstan) as *E. dione* aber. *tenebrosa*. However, he explicitly stated that these specimens did not merit subspecific status (p. 139). The name *tenebrosa* is now appropriately used by snake keepers to describe this color morph. Therefore, we argue that neither *niger* nor *tenebrosa* should be recognized as valid taxonomic entities at the subspecies level.

The Chinese lineage, first identified by Hofmann, Fritzsche & Miehe (2016) from southwestern central China, is deeply divergent (5.6–6.7% for *COI*) and likely of Late Miocene origin. This level of divergence typically corresponds to species-level distinction in Palearctic snakes (Wagner et al., 2016; Freitas et al., 2020; Kornilios et al., 2020; Jablonski et al., 2019; Jablonski et al., 2023). A DNA barcoding study of Chinese snakes (Wu et al., 2023) also suggested a potential species status for this clade based on barcode gap analysis, like the case of *E*. *zoigeensis* (Huang et al., 2012). However, further morphological, ecological, and multilocus genetic (genomic) studies are required to resolve its taxonomic rank. Under similar criteria, the Korean clade, which diverged at the Pliocene–Pleistocene boundary and shows moderate divergence (2–3.2% in *COI*) from the members of widespread clade, would also appear to warrant recognition as a distinct subspecies of *E. dione*.

Although matrilineal historical patterns tracked by mitochondrial phylogenies offer valuable clues about species’ dispersal history, relationships inferred with mtDNA might misrepresent organismal phylogeny for a number of reasons (Sloan, Havird & Sharbrough, 2017; Dufresnes, Poyarkov & Jablonski, 2023; Wüster, 2025). In our study, it was also not possible to achieve a comprehensive resolution of relationships across this recently radiated widespread species using only three mtDNA markers (constituting a 2,000 bp alignment). An analysis of the variability levels within each marker in the dataset revealed that 12S rRNA was relatively conservative and thus less informative for analysis at the intraspecies level in *E. dione*. The use of nuclear DNA markers in further studies of this species is essential to confirm observed patterns and refine phylogenetic resolution. In this context, application of genome-wide methods like RAD-seq would be beneficial over sequencing of several single copy nuclear markers (Dufresnes, Poyarkov & Jablonski, 2023), which often are not appropriately phylogenetically informative in snake species delimitation studies (reviewed by Wüster, 2025), and especially at intraspecies level (*e.g.*, Asadi et al., 2019; Jablonski et al., 2019; Jablonski et al., 2024).

## Conclusions

We conducted the first broad-scale phylogeographic study of the widespread steppe rat snake *E. dione* and found that the most active vicariant diversification in the Western and Eastern Palearctic occurred around the climatic and environmental transitions in the Mid-Pleistocene. While this cladogenesis occurred simultaneously across the species’ range, allopatric diversification in the Western Palearctic resulted in a greater number of phylogeographic lineages. This could be linked to a higher number of suitable refugia associated with the low-elevation river valleys of the Turan region in Central Asia. Spatial patterns of mtDNA variation also identified areas of post-LGM dispersal and secondary contact zones of several lineages. Although our study represents the most comprehensive phylogenetic analysis of *E. dione* to date, substantial gaps remain across its vast distribution. Targeted further sampling in Transcaucasia, Alborz Mountain region, Afghanistan, Uzbekistan, and central Kazakhstan and especially China is essential to better understand the species’ dispersal pathways and historical biogeography. Moreover, resolving the subspecific taxonomy of *E*. *dione* will require the inclusion of additional molecular markers and genome-wide data. This study thus provides an important first step and a foundation for future research, ideally conducted through broader international collaboration.

##  Supplemental Information

10.7717/peerj.20351/supp-1 Supplemental Information 1 Sequences used in the study

10.7717/peerj.20351/supp-2 Supplemental Information 2 Time-calibrated tree with all outgroups shown Numbers with branches indicate mean estimated node ages (in millions of years) and, together with blue bars, 95% highest posterior densities of the estimated node ages. Calibration points are highlighted with pink bars.

## Additional Information and Declarations

### Field Study Permissions

The following information was supplied relating to field study approvals (*i.e.*, approving body and any reference numbers): Field experiments were approved by the Ethics Council of M. Utemisov West Kazakhstan University (No. 2480; date of approval 4 September 2023), by the Ministry of Ecology, Environmental Protection and Climate Change of the Republic of Uzbekistan (No. 3310-8464-f6c5-b33e-c208-2408-8455/2023-09-05).

### DNA Deposition

The following information was supplied regarding the deposition of DNA sequences: The raw sequence data are available at NCBI: PV653725 to PV653803, PV657704 to PV657788, and PV660112 to PV660157.

### Data Availability

The following information was supplied regarding data availability: The data is available in the Supplementary File. The raw sequence data are available at NCBI: PV653725 to PV653803, PV657704 to PV657788, and PV660112 to PV660157.
