# Supplementary material for: Diversification in the steppe rat snake Elaphe dione (Pallas, 1773) coincides with the Mid-Pleistocene climatic transition of Eurasia
Source: PeerJ. 2026 Feb 2;14:e20351. doi: 10.7717/peerj.20351 (PMC12875252; doi:10.7717/peerj.20351)
Supplement: Supplemental Information 2 — Numbers with branches indicate mean estimated node ages (in millions of years) and, together with blue bars, 95% highest posterior densities of the estimated node ages. Calibration points are highlighted with pink bars. [file peerj-14-20351-s002.pdf]

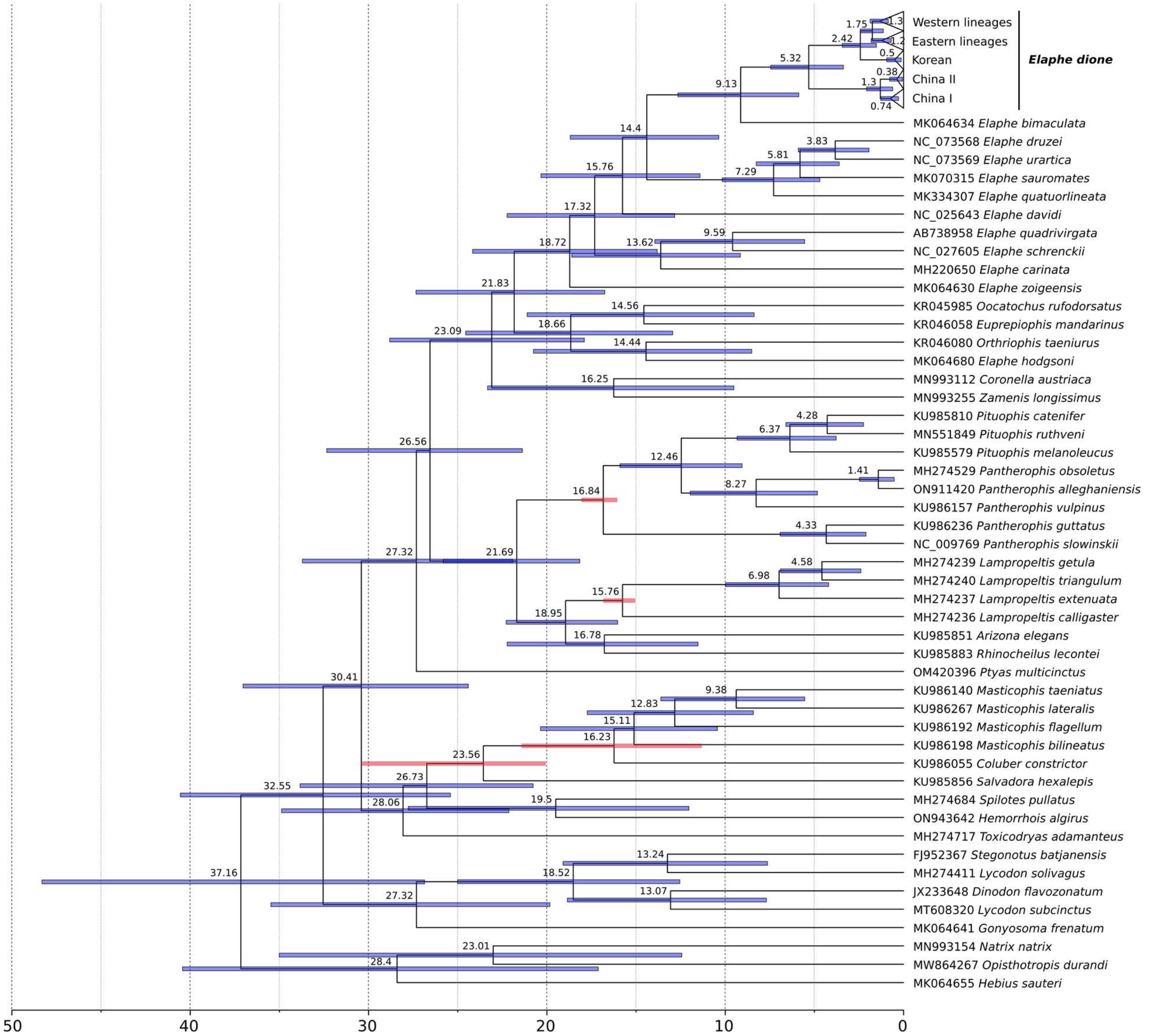

Figure S1. Time-calibrated tree with all outgroups shown. Numbers with branches indicate mean estimated node ages (in millions of years) and, together with blue bars, 95% highest posterior densities of the estimated node ages. Calibration points are highlighted with pink bars.
